# The interrelatedness of error prevention and error management

**DOI:** 10.3389/fpsyg.2023.1032472

**Published:** 2023-04-28

**Authors:** Connie A. Van der Byl, Harrie Vredenburg

**Affiliations:** ^1^Bissett School of Business, Mount Royal University, Calgary, AB, Canada; ^2^Haskayne School of Business, University of Calgary, Calgary, AB, Canada

**Keywords:** error management, error prevention, failure, qualitative study, environmental regulations

## Abstract

We study errors in organizations to understand and ideally prevent them from reoccurring. In this study we examine mistakes made as an oil company adopted new technology to access untapped reserves. We find that a pre-existing error management culture (EMC) dominated in the organization while error prevention measures were deficient. This is surprising given the complexity of the business and the importance of safety. We show that a balance between error prevention and error management is difficult to achieve owing to the contradictory nature of these approaches. While the extant organizational error literature identifies the complementary aspect of error prevention and error management it does not consider their interrelatedness–how one affects the other. We find that the dominating error management culture at Suncor Energy contributed to error prevention processes that were misapplied, informal or absent. This highlights the need for deliberate examination of error approaches especially as the business context shifts.

## Introduction

The causes of disasters in the oil and gas industry are often linked to errors. In the case of the BP Macondo Deepwater Horizon explosion, the National Commission’s (2011) report to the President contends that the disaster could have been prevented and that identifiable mistakes had been made by firms involved in the operation. Farther back in memory, the Exxon Valdez oil spill was attributed to human error. These events are often rightly or wrongly publicly linked to the low cost, corner cutting, “greediness” of oil companies. Certainly, oil and gas companies focus on reducing costs to increase margins in this commodity industry. However, the errors associated with disastrous outcomes have more complicated underlying causes including, but not restricted to, questionable cultures of safety and poor regulatory oversight (National Commission, 2011). This industry then provides an interesting and fruitful context for studying errors–especially given the potential for errors to lead to environmental and social harm. Much can be learned from organizational failure, and it remains a relatively untapped source of empirical data in management literature (Gino and Pisano, 2011; Hoffman and Jennings, 2011; Tinsley et al., 2011; Bansal and Hoffman, 2012).

Typically, in high-risk organizations (HROs), like those in the oil industry, error prevention is emphasized (Cowley et al., 2021), acknowledging the potential for significant errors. Error prevention approaches tend toward routines, standards, and controls. There is, however, an acceptance that even with stringent error prevention approaches, mistakes and failures can ensue. Ideally, error prevention is developed along with error management approaches that are adaptive, flexible, and executed during and after an unanticipated error occurs. Balancing these two contradictory yet complementary approaches is challenging (Van Dyck et al., 2005; Goodman et al., 2011; Frese and Keith, 2015; Lei et al., 2016).

In this paper, we examine mistakes that occurred at Suncor Energy, Canada’s largest integrated oil company, and that led to environmental noncompliance charges and a fine. Through unique access granted by a creative sentencing order we obtained candid observations and reflections on the mistakes leading to the fine from key personnel in the company. Creative sentencing in Alberta was developed in the late 1980s and allows prosecutors to use alternative measures to a traditional fine when prosecuting firms or individuals who fail to comply with environmental regulation. This creative sentence order, imposed by the Alberta provincial court, required Suncor to both fund and participate in a research project.

A single case study approach is used to facilitate in-depth study of the organizational errors that Suncor experienced. Interviews were conducted with 50 Suncor employees and 21 stakeholders from outside the organization. We used qualitative data analysis methods including iterative analysis, sense making, and a review of the extant literature. This led us to an empirical assessment of the research question: How are error prevention and error management interrelated? (Van Dyck et al., 2005; Goodman et al., 2011; Frese and Keith, 2015; Lei et al., 2016).

We find that error management was emphasized in this high-risk firm. The error management culture (EMC) was characterized by attitudes of moving quickly and fixing problems later. This is surprising given the risks inherent to the business and the high level of government regulation. The challenge of this approach became salient in a period of unprecedented industry growth owing to high commodity prices. In this context, the firm endeavored to move quickly in new technology adoption with associated mistakes and environmental infractions. Our findings contribute to the extant literature on organizational error by showing the need for a balance between error prevention and error management and the interrelatedness of these seemingly divergent yet complementary strategies.

## Understanding errors

Before considering error prevention and error management and their interrelatedness it is helpful to revisit the definitions of errors and associated concepts.

### Errors, mistakes and failure

Errors are unintended departures from what is planned or expected or incorrect actions owing to a lack of knowledge (Frese and Keith, 2015). Errors are distinct from violations, mistakes, and failure. Reason (1990) links the distinctions to intention. A violation has an associated intent or deliberateness (Frese and Keith, 2015). Failures are often a combination of errors and have negative organizational outcomes. They differ from errors in that not every error leads to failure (Dahlin et al., 2018). Errors can be detected and addressed before contributing to failure (Frese and Keith, 2015). Reason (1990) further categorizes the term error in connection to intent. Slips and lapses are unintended actions that do not proceed as planned. Mistakes carry intent in that the plan itself is inadequate (p. 17). And so, an error is a slip or lapse that is unintended and causes negative outcomes associated with deviating from the intended plan. A mistake, instead, stems from a faulty plan and so can be attributed to some avoidable action.

Mistakes are further categorized as stemming from rules-based or knowledge-based activities that go awry (Reason, 1990). Reason (1990) explains that it is human nature to search for existing, packaged answers to problems. And so, rules-based approaches to problems are common. These rules are simple to adopt and take less time and effort than knowledge-based activity which requires original thought to assess and address a problem. Rules-based solutions involve search for existing responses that appear to fit the problem at hand. Reason (1990) defines this as “similarity matching” and it is akin to constructs like “mirroring” and Kahneman’s (2011) “anchoring.” Simply put, we tend to want to deal with problems by applying solutions that have worked in the past and require application rather than development. As Reason (1990) explains this can lead to two types of errors: a misapplication of good rules; or an application of bad rules. These error types share traits with the psychology term “negative transfer” which has been adopted in the management literature on acquisitions (Finkelstein and Haleblian, 2002). Errors ensue when the similarity that was anticipated does not manifest. And so, the plan is wrong for the given situation either because the rules were bad to begin with or because the good rules are not applicable. Reason (1990) goes on to argue that knowledge-based processes can mitigate the risk of these rules-based errors through deliberate thought and consideration. A knowledge-based approach requires more effort but considers more deeply the complexity of the problem, uncertainty, ambiguity and the dynamic context. Typically, knowledge-based activity is used only when rules-based approaches have successively failed. Knowledge-based operations can suffer from bounded rationality and limited information or knowledge. These challenges occur when various psychological factors like confirmation bias, overconfidence and others prevent acknowledgement of these limitations to problem solving.

### Error prevention vs. error management

If intended actions leading to errors can be avoided, then error prevention can occur. Similarly, if we accept that some errors are unintended and unavoidable then error management is required. These are two distinct approaches to dealing with errors. Lei et al. (2016) offer that, “error prevention works by emphasizing routines, standardization, and control, while error management encourages adaptation, flexibility, and improvisation” (p. 1329). Goodman et al. (2011) define this as resiliency. While Cowley et al. (2021) deem it adaptation with error management requiring vigilance and situational awareness (p. 50). Lei et al. (2016) argue that there are three phases to effective error strategies: before, during and after. Error prevention occurs ex ante or before the error occurs while error management takes place ex post or during and after the error occurrence. During requires error management in real time action to detect, report and correct the error. After the error occurs, management approaches enable learning (Van Dyck et al., 2005). In this way error management has two elements: a quick response (damage control) and approaches to learn and prevent future errors with some scholars emphasizing the former (Goodman et al., 2011; Lei et al., 2016) and others the latter (Van Dyck et al., 2005; Frese and Keith, 2015). Van Dyck et al. (2005) extend definitions of error management to include a preventative approach through learning from errors.

### Complementary error prevention and error management approaches

The actions associated with error prevention and error management are contradictory. The former requires formal and deliberate standards, rules and controls to be established while the latter relies on informal improvisation, flexibility and adaptation. And yet, these contradictory approaches are complementary. That is, both work in service of mitigating the potential impact of errors (Lei et al., 2016). Errors cannot be completely prevented (Frese and Keith, 2015). Hollnagel (2009) describes “normal accidents” as unavoidable and occurring in complex systems. Any new action, like innovation, is likely to be error prone. However, this should not encourage trial and error approaches (Frese and Keith, 2015). Despite the unavoidable potential for errors, error prevention is a critical approach especially in complex or High-Risk Organizations (HROs). The medical and energy sectors are labeled safety industries and include HROs. Given the risk and the potential impact and scale of error these organizations tend to focus on error prevention. Several authors argue that while this emphasis on error prevention is warranted, error management strategies must co-exist. In this way, when an error inevitably occurs approaches are in place to address the error, mitigate its impact and learn from it moving forward (Frese and Keith, 2015).

The extant literature identifies error prevention as dominant, especially in high-risk industries, and highlights the value of error management that is responsive to unexpected errors but also fosters learning. In this way the value and complementary nature of both error prevention and error management is argued. In our study, we extend this research by examining a case in which error management, especially in responsiveness to errors rather than learning from errors, is dominant while error prevention is lacking. This overreliance on error management to the detriment of error prevention leads to organizational failure in the form of an environmental noncompliance. Through this work we show that beyond being complementary, error prevention and error management are interrelated–that is the presence or absence of one affects the other. Overreliance on error management increases organizational stressors. Our findings augment contentions that error prevention without error management can lead to longer term organizational challenges when the context changes and existing prevention measures are no longer sufficient (Van Dyck et al., 2005).

There is growing interest in the phenomenon of organizational errors, but key aspects remain understudied in “real” organizational settings. In particular, there is a need to study how contradictory priorities can affect error occurrences. An important contradiction may exist between error management and error prevention. There may be a conflict in prioritizing these two distinct approaches simultaneously. And yet, integration of the two error approaches is theoretically appealing to reducing errors.

Despite the value of having both approaches in place, integrating the two is challenging. We can accept that pursuit of error prevention in HROs may be insufficient when unexpected errors occur. We know less about an over reliance on error management. Lei et al. (2016) describe an error management culture where fixing errors is the focus. However, the implications for error prevention when an error management culture dominates is not clear. Further, while there is agreement on the complementary nature of the two approaches their interrelatedness is not considered. Possibly, the challenge of integrating the two strategies within organizations is tied to their relatedness. We explore this as we consider the mistake that occurred at Suncor Energy.

## Methods

We use a qualitative single case study research method to study the phenomenon of organizational error. The single case study approach allows for in-depth insights to be developed through analysis. Data is sourced predominantly from interviews with Suncor employees as well as individuals from outside the organization. We followed a grounded theory approach (Glaser and Strauss, 1967), not driven by *a priori* theoretical constructs. A grounded theory approach ensures that the data provides the answer to the research question rather than theory being the driver. To address concerns regarding the rigor and procedures applied to qualitative research (Parkhe, 1993; Eisenhardt and Graebner, 2007; Yin, 2009), data analysis procedures as prescribed by Miles and Huberman (1994) were used. Iterative review of the data and the extant literature led us to the research question of how error prevention and error management are interrelated.

### Context

Alberta, Canada has the third largest reserve of oil in the world, behind Saudi Arabia and Venezuela. Much of that oil reserve takes the form of oil sands; a mixture of bitumen and sand. Bitumen is a heavier oil about the consistency of molasses. Given this higher viscosity, extraction of bitumen requires more energy than conventional oil production methods. About 20% of the oil sands are accessible *via* surface mining techniques. This involves using shovels and trucks to extract and transport the bitumen to a processing facility. Like other open pit mining operations this results in removal of overburden and the creation of tailings ponds. The remaining 80% of bitumen in Alberta is below surface.

New technology called steam assisted gravity drainage (SAGD) was developed through an industry and government collaboration in the 1990s to access subsurface bitumen in the oil sands. This technology relied on horizontal drilling techniques which were also newly developed. The oil and gas industry for more than 50 years had relied on simple vertical drilling to access reserves. Horizontal drilling allowed drilling to occur vertically and then extend horizontally thereby enabling access to more of the product and reducing the surface footprint. SAGD technology was based on the injection of steam into the reservoir to effectively loosen or melt the heavy bitumen facilitating extraction. While one horizontal well injected steam the other would draw the bitumen from the reservoir to the surface. Creating the steam required heating water, typically with natural gas. The process was therefore costly but unleashed vast new reserves.

In the late 1990s, as oil prices began their meteoric rise, Suncor expanded its core oil sands mining business into new development through technology adoption. Commercialization of this *in situ* thermal recovery technology (or SAGD) occurred at a site called Firebag. Suncor had first applied SAGD technology at a pilot plant in Burnt Lake, a separate geographic location, in 1997. In 1999, Suncor began work on the design of the Firebag project. At the time, there were no other commercial SAGD operations in the Athabasca area. This expansion from traditional surface mining techniques to new below ground extraction technology was not without its challenges to Suncor, both operational and environmental.

In April of 2009, Suncor was fined a record $675,000 for two environmental offenses (Court Order, 2009). The company had failed to install pollution control equipment (a Vapor Recovery Unit or VRU) promised in the application for approval and failed to bring the missing equipment to the attention of the Alberta regulator in a timely manner. As a result of environmental infractions incurred by Suncor, a creative sentencing project was established to facilitate a regulatory compliance research project. Through unique access granted by the creative sentencing order we have candid observations and reflections on the errors leading to the fine from key personnel in the company. Creative sentencing enabled three faculty members and two PhD students to gather data from Suncor. Researchers divided into two teams. The first team focused their data gathering on regulators and the application of institutional theory. All researchers worked together to share insights from data collection and to validate findings. The authors of this paper interviewed Suncor employees (see Table 1) and were concerned with the issue of organizational failure from a strategic management perspective.

**Table 1:** Interview statistics.

Total number of interviews	71
Total number of informants	62
Interviews in Suncor	50
Suncor informants	41
Executive level (VP and above)	10
Director level and below	31
Interviews with regulators, legal and crown	14
Other interviews–industry players and NGOs	7

### Data collection

Gummesson (1991) emphatically contends that access to reality is the number one challenge facing business researchers (p. 11). Canada’s energy industry is competitive with many companies of varying sizes. This presents both opportunities and challenges to accessibility. Suncor data was made available *via* the creative sentencing order. The unique nature of the creative sentence was such that Suncor was compelled to share information with the research team. As a senior executive with Suncor commented, “it’s unusual not to worry about being candid for fear of legal action because in this case the legal action has already taken place.” Also, given that Suncor executives agreed to this creative sentence as part of a negotiation with the Crown, there was strong support for the research project from the top of the organization. This was beneficial in eliciting responses from targeted informants and also in obtaining administrative support in organizing interview logistics.

#### Interview data

In Suncor, our interviews started at higher level executives and snowballed to management and frontline employees. Initially, we spoke to executives either involved in the noncompliance or in responding to it *via* transformation of systems and culture. These executives then identified lower level employees who were similarly connected to the noncompliance or to the execution of change initiatives.

From September 2009 to January 2011, a total of 72 interviews were conducted, 51 within the organization and 21 interviews with external stakeholders. The final dataset contained 71 interviews as we did not receive consent to use one of the Suncor interviews. Most of the interviews were conducted in person in Calgary. All but two of the Suncor interviews were conducted by pairs of researchers to enhance the reliability of the findings through corroboration. A semi-structured approach was used for interviews that lasted between 30 and 90 min with the average interview time being 60 min. A panel interview was conducted on site at Firebag, north of Fort McMurray. Inaugural questions asked informants to provide background on their education and career trajectory up to their current role in the organization. Next, they were asked to recount their involvement or knowledge of the infractions that occurred at Suncor’s Firebag project. Interviewees who did not have firsthand knowledge of the incidents provided their understanding. These participants were typically involved in the development of systems and initiatives to improve Suncor’s performance in light of the Firebag and other incidents. All interviews were recorded on audio tape and transcribed by a third party. These 71 interviews resulted in more than 1,500 pages of interview transcripts. Initially, we analyzed transcripts for themes emerging in the data. Specific interview questions asked interview participants for the insights on why the environmental noncompliance charges were incurred and what was being done to remedy their causes. In recounting issues around the noncompliance, insights on deficiencies in processes, culture and the pace of expansion were provided. We stopped interviewing when these key themes repeated frequently, thereby bringing us to theoretical saturation. Also, we had contacted all individuals identified as critical to helping develop our understanding of the causes of the infractions. Data collection was governed by the Conjoint Faculties Research Ethics Board (CFREB) at the University of Calgary. We analyzed data using the computer program, Atlas.ti.

#### Secondary data

We triangulated primary data *via* secondary data sources. These sources included: media reports, government publications and reports, NGO reports, corporate archived data, analyst reports, consultant reports and court documents. These sources were particularly relevant in fact checking data provided by informants and in corroborating informant assessments of industry growth and market velocity. In addition, consultant reports and court documents provided corroboration and validity assessments for our findings. Court documents cite insufficient compliance assurance systems as leading to prosecution. A third party audit found inadequacy with the management of change process, informal compliance and integration processes, ineffective audit implementation, absence of compliance culture, incomplete pilot data and rapid growth as contributing to compliance and operational issues at Firebag (CH2MHill, 2008).

### Data analysis

A grounded theory approach was used, with open coding resulting in 152 codes. We applied Miles and Huberman (1994) approaches to categorize these codes. We determined that 12 codes were out of scope and 55 were of a low frequency. We thus reduced the number of codes to 85 for data analysis. As key themes emerged, we also began considering management theories in making sense of the data. Through this process we grouped codes into 11 categories. As analysis progressed, we saw a clear theme of a tension between required compliance processes and a propensity for Suncor to move quickly with only loose controls. This led us to see a connection to the literature on organization failure and errors. Through further data analysis iterations and consideration of the error literature we keyed in on the following categories:

- context.
- outcomes.
- processes.
- transfers.
- culture.

**Figure 1:**
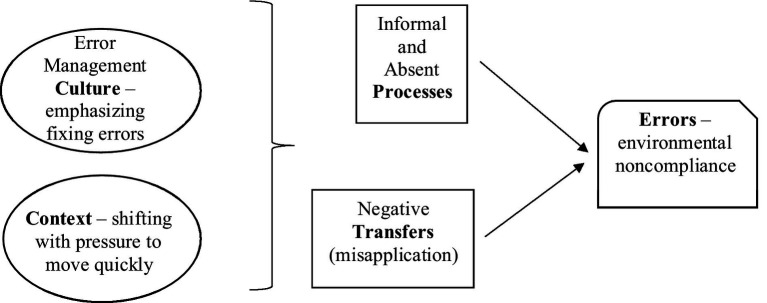
Empirical model.

The simple empirical model presented in Figure 1 shows further refinement of these categories to key variables and their interconnection. Culture and context led to informal/absent processes and negative transfers (misapplication) contributing most significantly to the error outcomes. Reviews of the extant error literature in tandem with data analysis refinement led us to the application of error prevention and error management concepts in our thinking.

Following engaged scholarship approaches outlined by Van de Ven (2007), findings were validated with external audiences through a technical industry conference presentation (Bowen et al., 2010a) and through a 1 day, crown mandated Knowledge Forum (Bowen et al., 2010b). Both events were held in downtown Calgary with significant Alberta oil and gas industry audience representation. The Knowledge Forum was held to fulfill obligations of the Creative Sentence Order to share findings with the industry and to offer an opportunity for learning. At this Forum, a panel session was held that included a Suncor executive and a high-level regulator. Through these sessions our findings were validated.

## Findings

We draw on interview participant details and secondary data to outline the events leading to the infractions. We find that a culture of error management superseded error prevention approaches at Suncor. That culture emphasized speed, informality and addressing mistakes and problems later. Further exacerbating this culture was external pressure to move quickly in the pursuit of growth. As represented in Table 2, our data analysis shows operational and regulatory processes as misapplied, informal, or absent and contributing directly to the environmental infractions or errors at Suncor.

**Table 2:** Processes contributing to mistakes.

Theory (Reason, 1990) of mistakes	Contributing factor	Infraction outcome	Representative quote
Rules-based misapplication of good rule–similarity.	Negative Transfer: Misapplication of *operational process*: Transfer of operational knowledge not applicable to new technology	Based on other similar projects expectation that VRU would not be needed–removed from design.	And so that’s been a key finding for. Equipment that fouls in one region fouls very differently where we are, so heavy oil is heavier, it’s stickier, it messes things up much more than you would see in an Imperial-Cold Lake-type facility. So it’s figuring out what’s the right instrumentation to measure certain things and then how you control that.
Rules-based misapplication of good rule–similarity.	Negative Transfer: Misapplication of *regulatory process*: Transfer of regulatory rules and knowledge not applicable to new technology.	Did not communicate and comply with appropriate regulatory body–failure to disclose.	So we transferred that same thought process and knowledge to Firebag and none of us understood the role of the Energy Resources Conservation Board (ERCB) at that point in time in these *in situ* facilities. We were thinking of it is an oil sands facility, right, far as we are concerned it is. ERCB looks at it very differently.
Rules-based bad rule–encoding deficiency	Informal *integration process*	Project integration between regulatory and design was informal contributing to the VRU being removed from the design but remaining in the application.	So without those interfaces really being properly understood without clarity of accountabilities on end to end project execution, which includes ultimately the operations handing off these things a recipe for disaster.
Rules-based bad rule–encoding deficiency	Informal *regulatory application review process*	Regulatory application review process prior to submission informal leading to VRU included in application but not in design.	I do not think that they formalized it enough. I think we are doing a much better job of it because having been involved in a few since then I know that the guy I work with on Firebag kind of understands that he will not submit anything until I say yes and give him back the information
Failure to execute a knowledge-based approach to the problem.	Absent *project implementation process*.	No project implementation process of the design not implemented and so no review of the final design by regulatory VRU removed from design.	In this vintage of time, we did not have a formalized Suncor project implementation process. I mean, the EPC firms may have had something themselves, but we were not doing formalized gate reviews and things like that. It was no give us a turnkey.
Failure to execute a knowledge-based approach to the problem.	Absent *approval review process.*	No formal approval review process to show the VRU in the approval but not on site leading to Failure to disclose.	Do not think we had to do any of that (approval review), really, on stage one. It wasn’t really clean up.

### A reactive error management culture at Suncor

Suncor informants use colorful language to describe the Suncor culture; adages like, “cowboy culture,” “pirate code,” “firefighters” and words reflecting a propensity for growth, action and informality. Interview participants exhibit a mixture of pride and pain as they describe an organization that valued fast action and fluidity rather than extensive analysis leading to bureaucracy and paralysis.

“I like the fact that Suncor has been a nimble company that can adjust. But you know if you don’t adjust or when you do adjust if you don’t have the processes to correct yourself or to catch up you’re going to run into issues like we had. So yeah you know I don’t want to see, as an employee, I don’t want to see us get burdened down in bureaucracy.” Suncor Employee

The dominant culture was characterized by informality, lack of process and standards, a drive to move quickly and acceptance to address problems later. Employees balked at process and preferred action. As one Suncor interviewee observed,

“Being the hero and you know very good at putting out fires and not so good at preventing them.”

There was an inherent impatience with process and procedure. A Suncor executive commented:

“Cultures that are different so that you know the analogy is Suncor is shoot, shoot, shoot aim and, Petro Canada is aim, aim, aim, aim, aim, shoot.”

The consequence of this culture was that challenges were taken on without extensive upfront planning and with the inherent expectation that problems could be fixed after they occurred. This led to the short pilot period that resulted in the misapplication of operational processes to Firebag. In addition, there existed a fast thinking logic and few formalized processes which influenced the activities in the Firebag design phase.

Like other companies in the commodity oil and gas industry, Suncor’s culture and associated systems valued cost saving and efficiency. Suncor employees and leadership were concerned with the environment and regulatory compliance. However, goals of cost and schedule, at times, were given higher priority than compliance. One candid observation by a Suncor interviewee was that,

“The frontline workers would be faced with a tough decision to make about do we cut things back or do we maintain our operation as it is knowing that we may be non-compliant and there wouldn’t be sufficient consequence after that, after a poor decision was made so that people took it as a signal that that was okay.”

Where clear processes and expectations did exist around operations, employees were compliant.

“There’s nobody in my time here, and nobody, I’d never seen anybody choose to be non-compliant. I mean, if people know what the rules are, they’ll do it.” Suncor Executive

These comments point to the importance of process related to this complex and regulated business. And yet, in the time of Firebag’s design, process and planning were lacking.

In Table 3 we connect interview descriptions of the Suncor culture with attributes of an Error Management Culture (EMC) characterized by real-time action, adaptation, flexibility and improvisation (Lei et al., 2016). We argue that prior to the SAGD technology adoption and expansion into a new business, the culture at Suncor supported moving quickly and fixing problems later. This approach was acceptable in the long-established oil sands mining business where processes had become institutionalized and the risk of significant error was minimal. In the new business there was greater uncertainty and higher potential for error. This organizational shift coupled with the dominating EMC increased the challenge of error prevention. Specifically, insufficient time was allocated to developing new knowledge-based processes. Instead rules-based approaches were developed that proved to be ineffective.

**Table 3:** Culture attributes.

Culture attribute	Link to infraction	Interview participant statement on culture
Informal–“*ad hoc*”	Informal application review process.	A lot of the way we operated was because we all worked together. I knew Hugh, he knew me. We, you know, we chatted all the time about stuff. That was the way a lot of stuff, and you know, fits would be sort of *ad hoc*. Here why do not you review the approval. Sort of all. um, cause I know who you are and I know what you do, so I think it would be a good idea for you to review this.
Fast pace	Process Misapplication.	You know, there’s no question we were moving quickly. But that is the way Suncor tends to do things. Is, you know, to do things relatively quickly. And if things need to be fixed afterwards, we’ll do that. The other model is you study it to death for years and years and generally still need to fix it at the end anyways. So, I’m not saying either model is perfect, by any means, but that’s generally the way we have done things.
Informal and “do” and do not worry about process.	Informal process and process absence.	So look at Suncor’s culture. It’s get it done, right? Move now. Process is missing; we just do, right? We’re not very rigid and very formal. It’s a very informal organization.
Do and fix problems later.	Misapplication, informal and absent processes.	Yeah, I would suggest uh Suncor, part of its very good success is this get-it-done, right. But it’s also now becoming apparent you know, it’s also part of the problem right…
Do and fix problems later.	Misapplication, informal and absent processes.	Which is get it done but oh yeah, by the way, now we got to fix all these mistakes, right. So when I look at uh I mean this is a whole different topic, but if you look at the amount of time and money and effort and money we have spent on fixing the Millennium Project build it’s huge
Firefighting–fix problems later.	Process absence.	And firefighting. And my sense is everyone up there firefights. I’m not saying they do not. But my sense is that they firefight a little less than we do.
No standards.	Informal process and process absence.	Standards are more like the pirate code… It’s more of a guide than a requirement.

### Error management damage control at Suncor contributes to rules-based mistakes

An internal Suncor review, external consultant review, Court documents and regulator informant data all corroborate the finding that insufficient, informal and absent processes were directly responsible for failure at Suncor.

#### Misapplication of processes

Adoption of SAGD technology at Suncor was informed by the oil sands mining business, Engineering Procurement and Construction (EPC) firms and experiences with the pilot project and similar technologies used by other companies. The regulatory approvals groups (Approvals) was staffed by people from the core business. Their knowledge and experience came from the well-established and simpler oil sands mining business and the newer but also well-developed natural gas division. Given the newness of the technology to both Suncor and the industry and given cultural and contextual factors that emphasized fast pace, there was an inclination to transfer existing processes to the new Firebag project. We find that this approach was directly responsible for both the missing VRU and the failure to disclose.

In developing the design of Firebag, the Project Team drew on their experience from earlier work assignments with Imperial Oil and from the short pilot project at Burnt Lake. Imperial Oil’s Cyclic Steam Simulation (CSS) was similar but not exactly like SAGD. Burnt Lake was located east of Firebag and the piloting phase occurred for only a short period of time. As a Suncor manager noted,

“We didn’t even pilot for too much of a length of time. There was a pilot … more towards the Cold Lake region … on so that they had some history in the field, but I think it might have been cyclic steam simulation.”

Reservoirs are sensitive to geography and react differently to production approaches depending on the formation.

“The facilities themselves are a big challenge, not even thinking about the reservoir which is you know even more mysterious”. Regulator

As a result, processes that worked at the pilot and were transferred to the commercial Firebag site were not applicable. A VRU was not required at the pilot as there were no H2S emissions. This informed the decision to remove the VRU from the Firebag design despite it being included in the regulatory applications. In addition, operational issues occurred at Firebag as unanticipated H2S was encountered.

“So you’ve got to remember we built stage 1 and stage 2 without ever having run a pilot up at that particular site and we had run a two well pier pilot down at Burnt Lake just north of Cold Lake. That’s where we thought we learned all our lessons and we went straight to two 35,000 barrel a day commercial schemes in stage 1 and stage 2 and guess what we got a lot of surprises we didn’t know were going to come to." Suncor Interviewee

Operational processes from other sites were misapplied at Firebag leading to “surprises” that culminated in the regulatory infraction and operational issues.

Regulatory knowledge and processes from the core oil sands business were transferred to the Firebag application process. However, SAGD facilities would report to a different regulatory body (Energy Resources Conservation Board or ERCB instead of Alberta Environment).

“There is a strong Alberta Environment focus down at Base Plant and up here I believe we’re, we have a lot more ERCB requirements than they would have down at Oil Sands Plant or the Oil Sands Mine. And just that lack of knowledge to begin with up here.” Suncor Manager

In addition, the regulatory requirements for Firebag were more complex and involved than had been experienced in the oil sands business.

“(We) were really surprised frankly at the amount of detail, and the amount of consultation, and the amount of work that had to go in ahead of time. And part of it is I think because of the scale of what you’re dealing with up there, it’s larger than a lot of other sites. And you know, we’re really familiar with regulatory approvals for batteries and they’re just so much smaller, it’s not nearly as involved as these very, very large applications.” Suncor employee

The oil sands regulatory processes that were transferred were misapplied. This led to challenges in filing the regulatory application and in knowing who to follow up and contact once the application was submitted and subsequently approved. Without the appropriate processes and contacts, Suncor did not understand the implications of self-disclosure and were fined accordingly.

“And so that was a key learning for us, how you report and what you report and the timeliness of that.” Suncor Interviewee

#### Informal processes

In the execution of any large-scale project there is integration between various organizational functions. In complex organizations an established project implementation process includes a formalized integration process. Typically, there would be stage gates associated with sign offs from various departments. This was informal at Suncor. Hand offs occurred between design and regulatory as well as regulatory and construction and operations. We find informal processes for integrating the pertinent functional areas as contributing to the infractions. A Senior Executive with Suncor admitted,

“The baton passing wasn’t – the exchange never led to a good interaction between the various groups who own compliance assurance and it’s not one group people don’t have a really good understanding of the integrated nature of some of these processes.”

Without a formalized project implementation process communication between the design and regulatory teams was informal and prone to error. This contributed directly to the VRU removal from the design not being communicated and being left out of the application.

Given the lack of formal project implementation process, a final review of the regulatory application should have been executed. However, in similar fashion, the *regulatory application review process* at Suncor was informal. The process involved a review of the regulatory application document by both the regulatory team and the design engineers to ensure that what was in the application was consistent with the design. This process was iterative and involved coordination of numerous revisions to the application. As explained by a Suncor informant,

“Oh so probably over the couple of months preceding this, hundreds. Because they would usually come back with … you know they’d review a section, she’d get some comments back – this needs to change or whatever. And the changes literally were … she would send them usually an electronic version of that section and most often they would then email her back all the changes that they wanted, but not in the document”.

One such revision was a request by a design engineer to remove gas blankets from the application as they were not necessary for the design since sour gas was not anticipated. References to gas blankets were removed from the application; however, because of a lack of technical experience on the Firebag regulatory team it was not clear that removal of gas blankets required removal of vapor recovery units (VRUs) as well. A gas blanket worked in tandem with a VRU. The gas blanket contained the emissions from the tank, in this case a produced water tank. While the VRU recovered those vapors for processing. In the end, the regulatory application contained reference to VRUs that were no longer in the design of the Firebag facilities. There was no formal sign off process where the design engineers might have flagged the inconsistency. As a result, the application was submitted with the VRU while it was no longer part of the design and would not be part of the facility that was eventually constructed. This was one of the charges against Suncor.

The Regulatory Team did not understand the distinction and relationship between gas blankets and VRUs. The Design Project Team did not review in detail the Application and compare it to Design. Effective communication and review between the disparate teams did not occur. Part of this can be attributed to the newness of the venture and associated technology both within the firm and in the industry. Regulations and governing regulatory bodies were not clearly established. The adoption and development of the new technology was not precedented and supporting structures were not in place.

#### Absent processes

In complex organizations an established *project implementation process* is typically executed to guide activity and to define accountabilities. Project milestones are aligned with stage gates and specific actions to move from one phase of a project to the next. This approach mitigates the potential for error and increases accountability. Prior to the Firebag infractions, Suncor relied heavily on EPC firms for project management. A Suncor Executive recalled,

“I think it was a period of time in which we had tremendous reliance on EPC firms to be the owners of not just the engineering design standards, but of quality and of - even of cost management. So, you know, you are really going to a turnkey type model on a green fields project without having – we didn’t have our major projects division in place either back at that time so that was a period of time in which we were still having the operating business try and act as project manager, as well as operator …”

This reliance on outside firms for project management and the absence of an internal project implementation process contributed to the VRU not being constructed since there was no stage gate requiring a check that the design and regulatory application were consistent.

The missing VRU might have been identified before charges were laid had Suncor implemented a formal *approval review process*. Once a regulatory application is approved by the regulator, an approval document is returned to the firm. In Suncor’s case that document was put in a filing cabinet and forgotten. Suncor was charged with failure to disclose because the noncompliance in not building the VRU while it was in the design was not reported from the point of commercialization, December 2003, until July of 2006. In the Alberta regulatory system, the regulator relies on a level of self-regulation. By Suncor not having a system in place to make that assessment they fell short on this self-regulation commitment and were charged accordingly.

### The resulting organizational failure: infractions

In 1999, an initial Design Basis Memorandum (DBM) was issued by an EPC firm. At the time, Suncor outsourced all of its design and construction work. The Design Basis Memorandum (DBM) included a vapor recovery unit (VRU) on the produced water tank. VRUs are used in upstream oil and gas to recover vent gas. It is a compression system that connects, in this case, to a fuel gas system. VRUs recover vent gas that would otherwise be emitted into the atmosphere or flared. As a result, they are important in meeting environmental regulations at production sites.

The DBM formed the basis for the Energy Utilities Board (EUB) and Alberta Environmental Protection and Enhancement Act (AEPEA) draft application. Later that year in December, a member of the Firebag Project Engineering Team at Suncor created personal notes that questioned the need for a gas blanket on the produced water tank. This question was conveyed to another Project Team member in a phone conversation on January 22, 2008. However, there was no formal documentation. In the February/March timeframe of 2000 the EPC contract for Firebag was awarded to a second EPC firm for Stage 1 construction. The initial EPC firm was subcontracted for process engineering.

From February to April 2000 the draft regulatory application was prepared by the Regulatory Approvals Team and reviewed by the Firebag Project Engineering Team. In the Facilities section of the draft a figure indicated a VRU from water tanks to the fuel system and a statement read “gas blanket on all water slop storage tanks.” On April 27, 2000 a Firebag Project team member provided the comment to the Approvals team that, “I would like to delete having gas blankets on all water tanks. The plan is to have a blanket on water tanks that have oil with water i.e. the skim tank, but for the treated and boiler feed water tanks, we would not’ necessarily blanket them. If we just say that we will blanket oily water tanks, I think it would be sufficient.” Root cause analysis documents provided to the research team by Suncor indicate that the “project wanted to save money.”

While the regulatory applications were being drafted, the Project Team worked on the design. A Process Flow Diagram (PFD) was changed on April 24, 2000 to enable “future provision for vapor recovery into the fuel gas system.” However, the vapor stream or tank were not specified. On May 5, 2000 the PFD was further revised to “add connections on the roof of the de-oiled PWT for VRU/gas blanketing.” On May 29, 2000 the Project Team Lead requested a meeting with the Approvals team to review the application in mid-June but there was no record of the meeting.

In May 2000 the application was submitted with the change made to reflect the request to remove gas blankets on all water tanks but still have vapor recovery on all water and slop tanks. Copies of the application were sent to the Project Team.

After receipt of the Approval Suncor did not complete a review to compare what was in the Approval to the actual design, construction and build of Firebag. Therefore, there was no internal observation that the VRU in the application was missing from the site. This led to the second charge by the Crown for failure to disclose.

## Discussion

The Creative Sentence Order that enabled this research required us to focus on determining what lead to the two environmental infractions at Suncor Energy. Specifically, what caused the environmental protection equipment (the VRU) from being included in the regulatory application for the project but removed from the design and actual facility construction. The second infraction was owing to Suncor’s failure to disclose this discrepancy to the Regulator. Given this mandate, our research question, interview observations and reflections, as well as secondary documents focus on what led to the infractions. In this way and connecting to Lei et al.’s (2016) work on the temporal dimensions of errors, our focus in this paper is ‘before’ the error occurs. That is, the period before the fines. According to Lei et al. (2016) in the ‘before’ phase, prevention strategies are used to defend against errors. In the ‘during’ phase, error management strategies are enacted, while in the ‘after’ phase, error management shifts attention to learning, sustainable performance, and innovation. This definition aligns with Van Dyck et al. (2005) who argue a control response and subsequent learning for future prevention are both part of an error management culture.

We find that the error prevention approaches at Suncor were deficient and contributed to the infractions. Our data points to misapplication, informal and absent processes as directly tied to the infractions. We further find that the culture at Suncor led to these incomplete processes. We draw on the extant literature to define that culture as focused on an error management culture (EMC). We contribute to the organizational error literature by showing the interrelatedness of this error management culture to the error prevention approaches. While the literature is clear on the contradictory and simultaneously complementary nature of error management and error prevention the interrelatedness of the two approaches and the associated implications are not empirically considered.

### Error prevention falls short at Suncor

There were problems with the processes in place that should have supported error prevention at Suncor as they adopted new extraction technology. The documents created and submitted as part of the regulatory application for design and construction of Firebag were important from the government’s perspectives in preventing errors–especially related to environmental performance. However, given the newness of the technology and the underlying culture at Suncor, there were problems with the regulatory and operation processes. As shown in Table 2, processes were misapplied, informal and absent.

The misapplication and absence of processes are identified as error problems by Reason (1990). He defines this as rules-based problems owing to either misapplication of good rules or application of bad rules. Misapplication of good rules is attributed to psychological tendencies for humans to seek out the familiar when faced with problems and to focus on similar common features rather than where aspects differ. This notion surfaces in the psychology and management literature as negative transfer (Finkelstein and Haleblian, 2002). A negative transfer occurs “when a prior event inhibits subsequent performance” (p. 36). This is more likely to occur when the two events share similar surface traits but have underlying differences (p. 36). Kahneman (2011) also addresses this through the concepts of representativeness, availability, adjustment and anchoring. Here also there is tendency to rely on that which is familiar when taking action.

Reason (1990) points out that a rules-based approach is expeditious and less appealing than the more onerous knowledge-based approach. According to Reason, the knowledge-based model to addressing mistakes is only adopted when successive rules-based failures have occurred. At Suncor, a culture of moving quickly coupled with the newness of the technology and external pressures to capitalize on high commodity prices all encouraged search for proximate and simple processes that could quickly be applied. These “good” rules or processes, however, proved to be inapplicable at the Firebag site resulting in mistakes and ultimately the environmental infraction. This tendency to move quickly and address problems later was part of Suncor’s culture and is also linked to the informal and absent processes that preceded the infractions.

### Interrelated error prevention and error management

We extend the notion of complementariness to interrelatedness. Not only do error prevention and error management approaches have the potential to work together to reduce the impact of errors but they are also reciprocal–one influences the other. In this way the two constructs are not divergent but rather interrelated. In our case study, an organizational culture that emphasizes error management influences a deemphasis of error prevention. This imbalance results in errors owing to deficient error prevention. This is particularly surprising given the company studied is in a complex, high risk industry. The implication of our assertion that error prevention and error management are not only complementary but interrelated is that each must be developed with consideration of the other. Too much emphasis on error prevention will make execution of error management approaches difficult because of the contradictory nature of activities involved. In the extant literature, we tend to accept that error prevention in high risk organizations dominates because of the significant potential for error and the belief that “prevention is better than a cure” (Hollnagel, 2009). However, perhaps it is the contradictory nature of prevention versus error management that results in the imbalance. In our evidence from Suncor Energy, we find that error prevention is deficient because of a dominating EMC that influences how error prevention is enacted.

To address this challenge, we suggest a deliberate assessment of error approaches and of the shifting business context. At Suncor, error prevention problems can be attributed to rules-based processes that were adopted based on their similarity to what was perceived as required. A knowledge-based assessment, though requiring more effort and time, might have highlighted the differences in the new business and facilitated the development of new error prevention processes. The Suncor case demonstrates how allocating more time to the ‘before’ stage could result in savings during and after a mistake occurs.

### Implications for practice

Today, organizations exist in turbulent contexts and face grand challenges (George et al., 2016). This may require quick responses in uncertain and unpredictable circumstances that could lead to failure. A strong foundation in error prevention can better prepare organizations for the unexpected. At the same time an error management culture can mitigate the impacts of failure and enable firms to learn from past mistakes. As our study suggests, an assessment and understanding of the culture and processes that allow both error management and error prevention to coexist has value. Balancing the focus on both these approaches simultaneously will serve the organization well but requires vigilance and situational awareness (Cowley et al., 2021, p. 50). We encourage leaders to make time for this continuous assessment. The alternative may be failure.

Organizations can learn from Suncor’s experience and dedicate time and effort to an evaluation of their culture and routines at any time, but especially before proceeding with new technology piloting and commercialization. Upfront, deliberate assessments and gap analysis can lead to identification of synergies and differences. Leaders are encouraged to consider the pre-existing culture and its implications for error management. Organizations that rely on simple, experiential, learn by doing approaches are cautioned to consider how those capabilities are enacted. Such an upfront systematic study may be challenged by an urgency to capitalize on growth opportunities. Companies that act in haste may face unanticipated challenges. Finally, organizations that interconnect with the natural environment are urged to be assured of their compliance routines even as they pursue higher level proactive and innovative environmental initiatives.

### Limitations and future research

This is a single case study and so prone to criticism for lack of generalizability. We note that the objective of the research is to generalize to theory and not to a larger sample (Van de Ven, 2007). This study was conducted in a high growth period in an industry that faces financial pressures to respond quickly to increasing product demand. Suncor was a company with a culture of moving quickly and fixing problems. This situation is not unique to the oil and gas industry; the potential exists for generalizability to other dynamic industries like high technology. Similarly, the environmental implications of a focus on error management to the detriment of error prevention exist in extractive industries like mining.

Further, the study is subject to recall bias given that events occurred from the late 1990s to 2007 and interviews began in August 2009 (Eisenhardt and Graebner, 2007). This risk is mitigated by the large and hierarchically diverse group of individuals interviewed.

In the future, this research study could be extended to a longitudinal process study by going back to Suncor and determining how new processes and error approaches have been executed and what the associated impact is in the organization. Research might focus on how Suncor integrated operations into project execution, formalized compliance processes and encouraged a new operational excellence culture to emerge. Further, there is an opportunity to return to the data and delve deeper into the issue and impact of culture at the time of the environmental noncompliance. In this way, contributions might be made to the literature on learning from failure and resilience.

## Data availability statement

The datasets presented in this article are not readily available because HREB restrictions. Requests to access the datasets should be directed to cvanderbyl@mtroyal.ca.

## Ethics statement

The studies involving human participants were reviewed and approved by Human Resources Ethics Board (HREB) University of Calgary. The patients/participants provided their written informed consent to participate in this study.

## Author contributions

All authors listed have made a substantial, direct, and intellectual contribution to the work and approved it for publication.

## Conflict of interest

The authors declare that the research was conducted in the absence of any commercial or financial relationships that could be construed as a potential conflict of interest.

## Publisher’s note

All claims expressed in this article are solely those of the authors and do not necessarily represent those of their affiliated organizations, or those of the publisher, the editors and the reviewers. Any product that may be evaluated in this article, or claim that may be made by its manufacturer, is not guaranteed or endorsed by the publisher.
